# Glycaemic Variability and C‐Peptide in Bangladeshi Children, Adolescents, and Young Adults With Diabetes: A Prospective Study

**DOI:** 10.1002/edm2.70347

**Published:** 2026-10-04

**Authors:** Ajmina Hasan Flabe, Bedowra Zabeen, Shatil Amirat, Jing Luo

**Affiliations:** ^1^ BADAS Paediatric Diabetes Care and Research Centre Diabetic Association of Bangladesh, BIRDEM Dhaka Bangladesh; ^2^ BRAC Dhaka Bangladesh; ^3^ Division of General Internal Medicine University of Pittsburgh Pittsburgh Pennsylvania USA

**Keywords:** Bangladesh, coefficient of variation, continuous glucose monitoring, C‐peptide, glycaemic variability, low‐ and middle‐income countries, paediatric diabetes

## Abstract

**Objectives:**

To characterise the magnitude of glycaemic variability and to examine the factors associated with it, with particular attention to residual beta‐cell function measured by fasting C‐peptide, in children, adolescents, and young adults with diabetes in Bangladesh.

**Design and Setting:**

Prospective observational study at the BADAS Paediatric Diabetes Care and Research Centre, BIRDEM, Dhaka, using 14 days of blinded professional continuous glucose monitoring (CGM). Participants were enrolled between March and November 2024.

**Participants:**

Ninety‐seven participants aged 1–25 years (68 with Type 1 and 29 with Type 2 diabetes) had at least seven valid CGM days and a paired HbA1c within 2 weeks of CGM initiation. Fasting C‐peptide was available for 46 participants.

**Main Outcome Measures:**

The coefficient of variation (CV) of glucose as the primary metric of variability; time in range, time above range, and time below range as secondary metrics; and independent predictors of CV on multivariable regression.

**Results:**

Mean CV was 39.4% (SD 9.2), and 59 of 97 participants (60.8%) were at or above the recommended target of 36%. CV was higher in Type 1 than Type 2 diabetes (41.9% vs. 33.3%). Lower fasting C‐peptide was independently associated with higher CV after adjustment for diabetes type, age, and insulin dose (−1.94 percentage points per ng/mL, 95% CI −3.35 to −0.53; model R‐squared 0.52), with a graded relationship across pre‐specified C‐peptide categories for CV, time in range, and time below range.

**Conclusions:**

Glycaemic variability substantially exceeds international targets in this setting. Fasting C‐peptide, available for fewer than half of the cohort, showed a graded association with variability that is best regarded as hypothesis‐generating; if confirmed in larger studies, it could offer a low‐cost means of identifying children at highest variability risk where CGM access is limited.

## Graphical abstract

In 97 Bangladeshi children and young people with diabetes, glucose variability far exceeded international targets. Lower residual beta‐cell function, measured by fasting C‐peptide, was associated with greater variability in a graded pattern. C‐peptide may help identify those at higher risk, though larger studies are needed to confirm this.

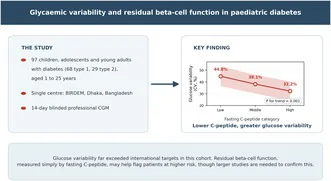

**Abbreviations**
BMI: body mass index
CGM: continuous glucose monitoring
CI: confidence interval
CV: coefficient of variation
HbA1c: glycated haemoglobin
HIC: high‐income country
LMIC: low‐ and middle‐income country
SD: Standard deviation
TAR: Time‐above‐range
TBR: time‐below‐range
TIR: time‐in‐range

## 1 Introduction

Continuous glucose monitoring (CGM) has fundamentally transformed the assessment and management of children and adolescents with diabetes by providing granular, real‐time glucose data that capture not only mean glucose levels but also the pattern and magnitude of glucose fluctuations throughout the day and night [1, 2, 3, 4]. Although previous monitoring approaches relied on periodic point estimates such as fingerstick glucose and HbA1c, CGM enables quantification of glycaemic variability, the dynamic rises and falls of glucose levels, which emerging evidence suggests represents an independent predictor of hypoglycaemia risk, glycaemic control quality, and potentially long‐term complications [5, 6, 7, 8]. Furthermore, these devices facilitate the detection of asymptomatic nocturnal hypoglycaemia, which remains a critical clinical concern in paediatric diabetes management [9, 10].

Glycaemic variability is typically quantified using several complementary CGM‐derived metrics. The coefficient of variation (CV), calculated as the standard deviation of glucose divided by the mean glucose and expressed as a percentage, is the most widely adopted measure of short‐term glucose fluctuation [7, 11, 12, 13]. International consensus guidelines recommend a coefficient of variation target of 36% or below [14].

In children and adolescents with diabetes, as values exceeding this threshold are associated with increased risk of hypoglycaemic episodes, particularly nocturnal hypoglycaemia [14, 15]. Time‐in‐range, time‐above‐range, and time‐below‐range provide complementary information on the distribution of glucose readings across the glycaemic spectrum and collectively characterise the quality of glycaemic control beyond mean HbA1c [16]. Additionally, the ‘time in tight range’ metric, representing readings between 70 and 140 mg/dL, has emerged as a refined parameter to assess optimal glycaemic stability in paediatric cohorts [17].

The clinical significance of glycaemic variability has been increasingly recognised in well‐resourced paediatric diabetes cohorts from high‐income countries, where multiple observational studies have linked elevated CV to higher rates of severe hypoglycaemia, diabetic ketoacidosis, and reduced quality of life [18, 19]. However, robust data characterising glycaemic variability and its clinical predictors in paediatric diabetes populations from low‐ and middle‐income countries remain sparse. This knowledge gap is particularly relevant for South Asia, where rapid scaling of CGM technology has begun but clinical guidance on the interpretation of variability metrics remains limited, and where factors such as medication adherence, dietary consistency, insulin formulation availability, and access to structured follow‐up may influence glucose fluctuation in ways that differ meaningfully from resource‐abundant settings [20].

Residual beta‐cell function, reflected by measurable fasting C‐peptide, has emerged as a biologically coherent correlate of glycaemic variability [21]. Preserved endogenous insulin secretion buffers postprandial glucose excursions through physiologic, meal‐responsive insulin release and supports counter‐regulatory glucagon responses that restrain hypoglycaemic swings, mechanisms that exogenous insulin therapy reproduces only imperfectly [22, 23]. Adult studies have demonstrated that higher C‐peptide correlates with lower CV and higher TIR, and recent paediatric data from high‐income countries have begun to extend this association to youth with Type 1 diabetes [24, 25]. However, C‐peptide's relationship with CGM‐derived variability metrics has not been characterised in a mixed Type 1 and Type 2 paediatric cohort from South Asia, where differences in dietary pattern, insulin formulation availability, monitoring frequency, and treatment intensification thresholds may alter the magnitude and clinical expression of variability in ways not anticipated by high‐income country data [26, 27, 28, 29].

Three intersecting gaps motivate this study. First, population‐level variability data from LMIC paediatric diabetes cohorts are absent from the literature; the 32%–37% CV range that informs international consensus targets derives almost entirely from well‐resourced cohorts using real‐time CGM, where the device's visibility to the patient may itself reduce glucose fluctuation through behavioural modification [30], Second, existing paediatric studies have often focused on Type 1 diabetes; characterising variability in a clinically realistic mixed cohort—where Type 2 diabetes is increasingly prevalent among adolescents in South Asia—is essential for developing regionally applicable guidance [31, 32]. Third, even where CGM is becoming accessible in LMICs, access remains intermittent and unequal [33]; investigating whether an inexpensive, routinely available test such as fasting C‐peptide can reliably stratify variability risk could provide clinical value for clinicians with limited access to continuous glucose sensors [34].

We aimed to characterise the prevalence and magnitude of glycaemic variability in a paediatric diabetes cohort in Bangladesh using blinded professional CGM—which eliminates the behavioural glucose modification inherent to real‐time devices—and to examine factors associated with elevated variability, with particular attention to residual beta‐cell function as measured by fasting C‐peptide.

## 2 Methods

### 2.1 Study Design and Setting

The study is reported in line with the STROBE guidance for observational studies. We conducted a prospective observational study at the BADAS Paediatric Diabetes Care and Research Centre, BIRDEM, Dhaka, Bangladesh, a national tertiary referral facility providing specialist paediatric diabetes care. Patients and families were consulted informally during the planning phase about the acceptability and burden of the monitoring protocol; they were not formally involved in the design of the study, the interpretation of results, or the preparation of this manuscript. Participants were enrolled consecutively during routine clinical review visits between March 2024 and November 2024. The study was approved by the Ethics Review Committee of the Diabetic Association of Bangladesh (Protocol No. BADAS‐ERC/EC/24/16) and conducted in accordance with the Declaration of Helsinki. Written informed consent was obtained from all participants aged 18 years and above and from parents or legal guardians of participants under 18 years. Age‐appropriate written assent was additionally obtained from participants aged 7 years and above.

### 2.2 Participants

Eligible participants were children, adolescents, and young adults aged 1–25 years with established Type 1 or Type 2 diabetes, attending routine clinical follow‐up at BADAS. Diabetes type was classified using a combination of clinical criteria and available biochemical markers. Type 1 diabetes was diagnosed based on: (a) clinical presentation with acute onset, ketosis‐prone phenotype, and requirement for insulin from diagnosis; (b) low or undetectable fasting C‐peptide (below 0.5 ng/mL) when available; and (c) age at diagnosis typically below 14 years. Type 2 diabetes was diagnosed based on: (a) gradual onset without ketosis, typically in adolescence; (b) elevated BMI and clinical features of insulin resistance; and (c) detectable or elevated fasting C‐peptide (0.5 ng/mL or above) when available. In this resource‐limited setting, autoantibody testing (GAD, IA‐2) is not routinely available, so clinical phenotype and C‐peptide were the primary classification tools. Exclusion criteria were: severe acute illness requiring hospitalisation, pregnancy, inability to tolerate sensor placement, or planned travel preventing sensor return for data download.

Of 120 participants initially enrolled, 23 were excluded because of missing or insufficient CGM data (fewer than 7 days of valid readings) or the absence of a paired HbA1c measurement within 2 weeks of CGM initiation, leaving 97 participants in the final analytic cohort.

CGM protocol: All participants underwent 14 days of blinded professional CGM using the Abbott FreeStyle Libre Pro iQ system (Abbott Diabetes Care, Alameda, CA, USA). The sensor was applied to the upper arm by trained clinical staff and remained blinded throughout the monitoring period, meaning participants could not view their glucose readings in real time. This approach eliminates behavioural modification associated with real‐time CGM visibility and provides a more representative estimate of true day‐to‐day glycaemic variability. Sensors were programmed to record interstitial glucose values every 15 min. After 14 days, participants returned to the clinic for sensor removal and data download. CGM data were extracted using the manufacturer's proprietary software and exported for analysis. A minimum of 7 days (70% of monitoring period) with valid sensor data were required for inclusion in the primary analysis. Sensor accuracy and calibration are factory‐set for the FreeStyle Libre Pro iQ system and do not require fingerstick calibration.

### 2.3 Data Collection

Demographic, anthropometric, and clinical variables were recorded at the time of CGM initiation from structured clinical records and direct measurement. Age, sex, diabetes type, diabetes duration, and current insulin regimen were extracted from medical records. Height and weight were measured using calibrated stadiometers and digital scales, respectively, with participants in light clothing and without shoes. BMI was calculated as weight in kilograms divided by height in metres squared. Total daily insulin dose was calculated as the sum of basal and bolus insulin doses averaged over the 7 days preceding CGM initiation, as reported by participants and verified against prescription records where available. Laboratory HbA1c was measured from venous blood samples collected within 2 weeks of CGM initiation using high‐performance liquid chromatography aligned with National Glycohemoglobin Standardisation Program (NGSP) accreditation standards, performed at the BIRDEM central laboratory.

Fasting C‐peptide was selected as the primary measure of residual beta‐cell function because it is inexpensive, widely available in routine clinical biochemistry laboratories across low‐ and middle‐income countries and does not require the stimulation protocols needed for more complex assessments of endogenous insulin secretion. Blood samples were collected after an overnight fast (minimum 8 h) on the morning of CGM sensor application. C‐peptide was measured in the BIRDEM central laboratory using a standardised chemiluminescent immunoassay (Siemens ADVIA Centaur; analytical measuring range 0.1–10.0 ng/mL; lower limit of detection 0.01 ng/mL; laboratory reference range 0.5–3.0 ng/mL for healthy adults). C‐peptide data were available for 46 of 97 participants (47.4%). The availability of C‐peptide testing was determined by logistical and resource constraints rather than systematic selection criteria. To assess potential selection bias, we compared baseline demographic and glycaemic characteristics between participants with and without available C‐peptide data; no significant differences were observed for age, sex, diabetes type, diabetes duration, BMI, HbA1c, mean CV, or TIR (all *p* > 0.70), supporting an assumption of effectively random missingness.

The primary outcome was coefficient of variation (CV), calculated by the CGM software as the standard deviation of all valid 15‐min interstitial glucose readings divided by the mean glucose and expressed as a percentage. Participants were classified according to whether their CV was at or above the internationally recommended threshold of 36% based on consensus guidelines from the International Society for Paediatric and Adolescent Diabetes [5].

Secondary outcomes were: (a) time‐in‐range (TIR), defined as the percentage of time with sensor glucose 70–180 mg/dL (3.9–10.0 mmol/L); (b) time‐above‐range (TAR), defined as the percentage of time with sensor glucose above 180 mg/dL; (c) time‐below‐range (TBR), defined as the percentage of time with sensor glucose below 70 mg/dL; and (d) mean sensor glucose, calculated as the average of all valid 15‐min readings over the monitoring period. These metrics align with international consensus recommendations for CGM data interpretation.

To explore dose–response relationships between residual beta‐cell function and glycaemic variability, participants with available C‐peptide data were grouped into three pre‐specified clinical categories using fixed C‐peptide cut‐offs. The category boundaries (low: below 0.2 ng/mL; middle: 0.2–1.0 ng/mL; high: above 1.0 ng/mL) were pre‐specified on clinical grounds rather than to produce equal‐sized groups, with the lowest category representing near‐complete beta‐cell depletion and the highest category representing relatively preserved endogenous insulin secretion.

### 2.4 Statistical Analysis

Sample size was determined pragmatically based on the availability of blinded professional CGM sensors during the study period, which were provided through collaboration with the University of Pittsburgh in association with the HumAn‐1 clinical trial. The target enrolment of 120 participants was consistent with prior blinded professional CGM studies in paediatric diabetes, which typically include 50–150 participants. With 97 participants, the study had reasonable precision to characterise a difference of this order in mean CV between diabetes types; formal power was not computed post hoc, and the confidence intervals reported below convey the precision of each estimate.

Continuous variables are reported as mean with standard deviation (SD) for approximately normally distributed data or median with interquartile range (IQR) for skewed data. Categorical variables are reported as number and percentage. The distribution of continuous variables was assessed visually using histograms and quantile‐quantile plots and tested statistically using the Shapiro–Wilk test.

Group comparisons for continuous variables used independent samples *t*‐tests when distributional assumptions were met (normality verified by Shapiro–Wilk *p* > 0.05 and similar variances confirmed by Levene's test) or Mann–Whitney *U* tests for non‐normal distributions. Categorical variables were compared using Pearson's chi‐squared test or Fisher's exact test when expected cell counts were below 5.

Univariable associations between clinical variables and CV were examined using Pearson correlation coefficients for normally distributed variables or Spearman rank correlation coefficients for non‐normal distributions. Variables with a univariable association at *p* < 0.20 were considered as candidates for inclusion in the multivariable model.

Multivariable linear regression was used to identify independent predictors of CV. Candidate predictors were selected based on clinical plausibility (diabetes type, fasting C‐peptide, age, total daily insulin dose) and univariable screening (*p* < 0.20). Diabetes type was entered as a binary variable (Type 1 vs. Type 2 diabetes, with Type 2 as the reference category). Fasting C‐peptide, age, and total daily insulin dose were entered as continuous variables. The regression analysis was restricted to the 46 participants with available C‐peptide data (complete‐case analysis). Because fasting C‐peptide also contributed to the classification of diabetes type, the two variables are partially collinear; the C‐peptide association is therefore interpreted as reflecting a gradient of residual beta‐cell function rather than an effect fully independent of how diabetes type was assigned, and its robustness was examined in a sensitivity analysis restricted to participants with Type 1 diabetes. Model assumptions were verified using residual‐versus‐fitted plots (to assess linearity and homoscedasticity), scale‐location plots (to assess constant variance), and normal quantile‐quantile plots of residuals (to assess normality). Cook's distance was calculated to identify influential observations. No major violations of regression assumptions or influential outliers (Cook's distance > 1) were identified.

To examine dose–response relationships between residual beta‐cell function and glycaemic outcomes, participants were stratified by C‐peptide category, and trends across ordered groups were assessed using the Jonckheere–Terpstra test for ordered alternatives.

Sensitivity analyses were conducted by (a) restricting the analysis to participants with Type 1 diabetes only, to assess whether the association between C‐peptide and CV was driven primarily by this subgroup; and (b) restricting the analysis to participants with at least 10 active CGM days (93 of 97 participants), to assess the robustness of findings to CGM adherence.

Missing data were handled using complete‐case analysis for the multivariable regression model (participants with missing C‐peptide were excluded from this analysis but included in all other analyses). We did not perform multiple imputation because the proportion of missing C‐peptide data (52.6%) exceeded the threshold at which imputation is generally considered reliable, and because missingness appeared to be unrelated to measured baseline characteristics.

All statistical tests were two‐sided with a significance threshold of *α* = 0.05. Analyses were performed using Python version 3.11.0 (SciPy, NumPy, Pandas, Statsmodels libraries) and R version 4.1.2 (base stats package and ggplot2 for visualisation). Data management and figure preparation were conducted using Python and R.

## 3 Results

Of 120 participants enrolled between March and November 2024, 23 were excluded because of missing or insufficient CGM data (fewer than 7 days of valid readings, *n* = 18) or the absence of a paired HbA1c measurement within 2 weeks of CGM initiation (*n* = 5), leaving 97 participants in the final analytic cohort. Their mean age was 12.3 years (SD 3.4), and 57.7% were female. Sixty‐eight participants (70.1%) had Type 1 diabetes and 29 (29.9%) had Type 2 diabetes. Ninety‐three participants (95.9%) completed at least 10 active CGM days, supporting the representativeness of the monitoring period.

Participants with Type 1 diabetes were younger (mean 11.1 vs. 15.0 years, *p* < 0.001), had lower BMI (19.0 vs. 26.7 kg/m^2^, *p* < 0.001), higher HbA1c (9.1% vs. 8.0%, *p* = 0.003), higher total daily insulin dose (28.4 vs. 22.1 units/day, *p* = 0.01), and substantially lower fasting C‐peptide (mean 0.4 vs. 2.3 ng/mL, *p* < 0.001) than those with Type 2 diabetes, consistent with the expected biological distinction between complete and partial beta‐cell failure (Table 1).

**TABLE 1:** Baseline demographic, anthropometric, and clinical characteristics of participants by diabetes type.

Characteristic	Type 1 diabetes (*n* = 68)	Type 2 diabetes (*n* = 29)	*p*
Demographic and clinical characteristics			
Age, years	11.1 (3.4)	15.0 (1.1)	< 0.001
Female sex, *n* (%)	39 (57.4)	17 (58.6)	0.91
BMI, kg/m^2^	19.0 (3.8)	26.7 (5.2)	< 0.001
Diabetes duration, years	3.8 (2.9)	2.4 (1.8)	**0.02**
Fasting C‐peptide, ng/mL^a^	0.4 (0.3)	2.3 (1.7)	< 0.001
HbA1c, %	9.1 (1.9)	8.0 (1.6)	**0.003**
Total daily insulin dose, units/day	28.4 (11.2)	22.1 (9.8)	**0.01**
Insulin regimen: MDI, *n* (%)	58 (85.3)	24 (82.8)	0.74
CGM wear ≥ 10 days, *n* (%)	65 (95.6)	28 (96.6)	0.81
CGM‐derived glycaemic metrics			
Coefficient of variation, %	41.9 (8.1)	33.3 (8.9)	< 0.001
CV ≥ 36%, *n* (%)	52 (76.5)	7 (24.1)	< 0.001
Standard deviation, mg/dL	46.8 (18.2)	31.4 (12.3)	< 0.001
Time‐in‐range, %	52.5 (13.8)	70.3 (16.5)	< 0.001
Time‐above‐range, %	36.6 (18.2)	19.6 (18.3)	< 0.001
Time‐below‐range, %	10.9 (8.9)	10.1 (12.3)	0.73
Mean glucose, mg/dL	168.4 (38.2)	140.2 (35.1)	< 0.001

*Note:* Data are presented as mean (SD) unless otherwise stated. *p*‐values in bold indicate statistical significance at *p* < 0.05. Group comparisons used *t*‐tests or Mann–Whitney *U* tests for continuous variables and chi‐squared tests for categorical variables.

Abbreviations: BMI, body mass index; CGM, continuous glucose monitoring; CV, coefficient of variation; HbA1c, glycated haemoglobin; MDI, multiple daily injections; TIR, time‐in‐range (70–180 mg/dL); TAR, time‐above‐range (> 180 mg/dL); TBR, time‐below‐range (< 70 mg/dL).

^a^ C‐peptide available in 46 of 97 participants (30 Type 1, 16 Type 2); patients with and without C‐peptide data did not differ significantly on primary outcomes (all *p* > 0.70).

Fasting C‐peptide data were available for 46 of 97 participants (47.4%): 30 of 68 participants with Type 1 diabetes and 16 of 29 with Type 2 diabetes. Participants with and without available C‐peptide data did not differ significantly on baseline demographic characteristics (age, sex, diabetes type, diabetes duration, BMI), HbA1c, or primary glycaemic outcomes (CV, TIR; all *p >* 0.70), supporting the assumption that C‐peptide missingness was effectively random with respect to measured characteristics.

### 3.1 Glycaemic Variability Burden

The mean CV across the full cohort was 39.4% (SD 9.2), with individual values ranging from 19.3% to 59.1%. Fifty‐nine participants (60.8%) had a CV at or above the internationally recommended target of 36%, indicating that suboptimal glycaemic variability was the norm rather than the exception in this cohort. This mean CV is substantially higher than the 32%–37% range consistently reported in well‐resourced paediatric diabetes cohorts from high‐income countries using real‐time CGM. This comparison should be read with some caution, because those cohorts largely used real‐time personal CGM, which tends to lower observed variability relative to the blinded monitoring used here.

Participants with Type 1 diabetes demonstrated higher variability than those with Type 2 diabetes across all CGM metrics (Table 1). Mean CV was 41.9% (SD 8.1) in Type 1 diabetes compared with 33.3% (SD 8.9) in Type 2 diabetes (mean difference 8.6 percentage points, 95% CI 4.6 to 12.6, *p* < 0.001). Seventy‐six percent of participants with Type 1 diabetes (52 of 68) had a CV at or above the target, compared with 24% of those with Type 2 diabetes (7 of 29, *p* < 0.001).

TIR was significantly lower in Type 1 diabetes (52.5%, SD 13.8) than in Type 2 diabetes (70.3%, SD 16.5, mean difference 17.8 percentage points, 95% CI 10.8–24.8, *p* < 0.001). TAR was correspondingly greater in Type 1 diabetes (36.6% vs. 19.6%, *p* < 0.001). In contrast, TBR was similar between groups (10.9% vs. 10.1%, *p* = 0.73), indicating that hypoglycaemia exposure was not confined to the Type 1 diabetes subgroup.

### 3.2 Univariable Predictors of Glycaemic Variability

On univariable analysis, fasting C‐peptide concentration showed a strong inverse association with CV (Spearman *r* = –0.39, *p* < 0.001), such that higher C‐peptide (reflecting greater residual beta‐cell function) was associated with lower glycaemic variability (Figure 1). Age showed a non‐significant trend toward lower CV in older participants (*r* = –0.18, *p* = 0.07). Sex, BMI, diabetes duration, and total daily insulin dose were not significantly associated with CV (all *p >* 0.20).

**FIGURE 1:**
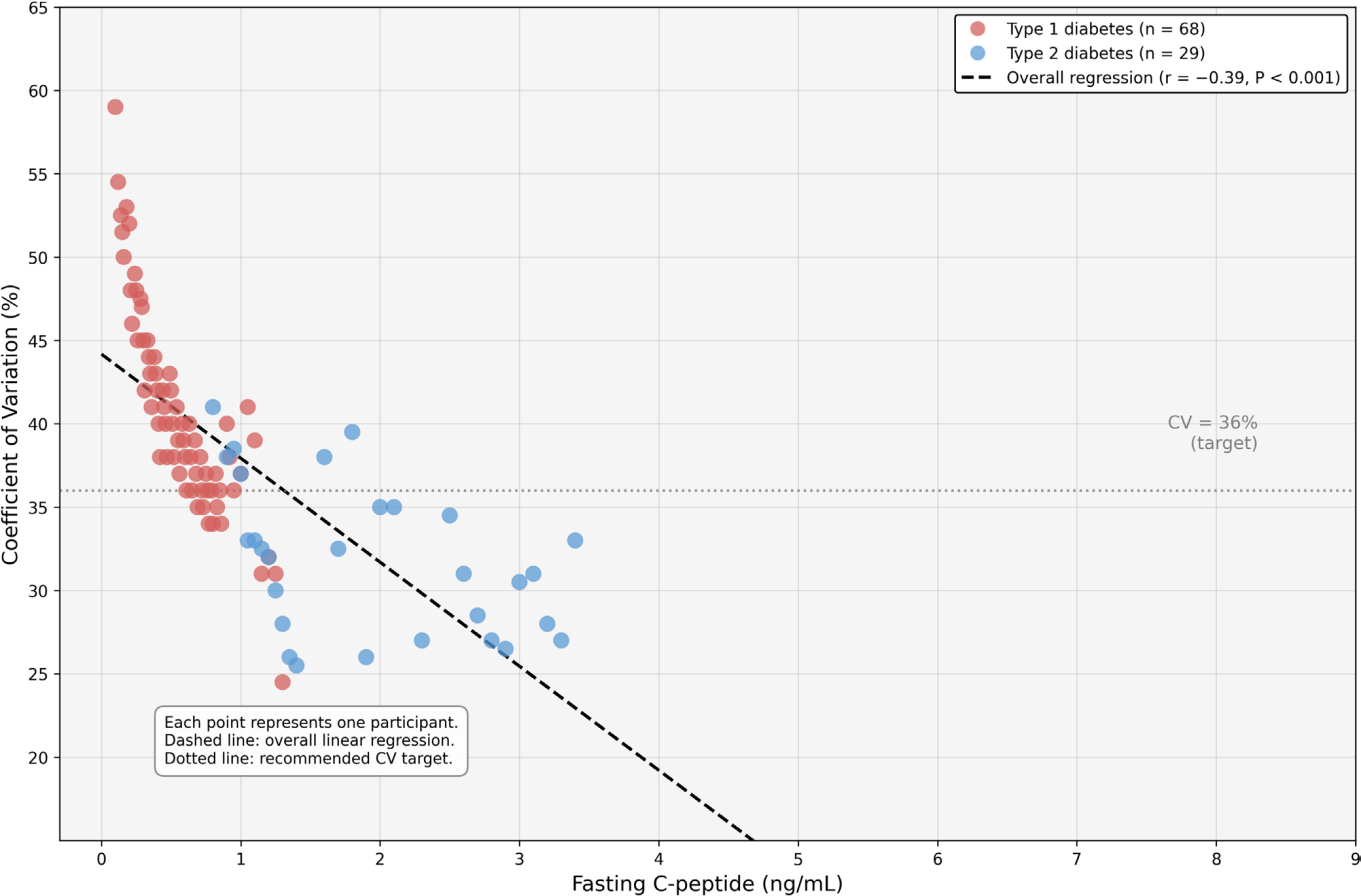
Glycaemic variability versus residual beta‐cell function by diabetes type. Scatter plot of coefficient of variation (CV, %) against fasting C‐peptide concentration (ng/mL) for 46 participants, stratified by diabetes type. Each point represents one participant. The dashed line represents the overall linear regression across both groups (Spearman *r* = −0.39; *p* < 0.001). The dotted horizontal line indicates the internationally recommended CV target of 36%. The shaded regions demarcate above‐target (red) and at‐or‐below‐target (green) variability zones. CV, coefficient of variation.

### 3.3 Multivariable Linear Regression: Independent Predictors of CV

In the multivariable linear regression model restricted to the 46 participants with available C‐peptide data, diabetes type and fasting C‐peptide emerged as independent predictors of CV (Table 2). Type 1 diabetes was associated with a 7.82 percentage point higher CV compared with Type 2 diabetes after adjustment for C‐peptide, age, and total daily insulin dose (95% CI 4.67–10.97, *p* < 0.001). Fasting C‐peptide retained a significant independent inverse association with CV, with each 1 ng/mL increase in C‐peptide associated with a 1.94 percentage point reduction in CV (beta –1.94, 95% CI –3.35 to –0.53, *p* = 0.008). Because diabetes type was itself defined in part using fasting C‐peptide, the two variables are partially collinear, so this association is best interpreted as a gradient of residual beta‐cell function rather than an effect fully independent of classification; its persistence within the Type 1 subgroup in the sensitivity analyses argues against it being solely an artefact of how type was assigned. Because C‐peptide was available for fewer than half of the cohort, these C‐peptide analyses should be read as hypothesis‐generating.

**TABLE 2:** Multivariable linear regression: Independent predictors of glycaemic variability (coefficient of variation).

Predictor variable	*β* coefficient	95% confidence interval	SE	*p*
Type 1 diabetes (vs Type 2)	+7.82	4.67 to 10.97	1.61	< 0.001
Fasting C‐peptide (per ng/mL increase)	−1.94	−3.35 to −0.53	0.71	**0.008**
Age (per year increase)	+0.21	−0.01 to +0.43	0.11	0.07
Total daily insulin dose	+0.04	−0.02 to +0.10	0.03	0.22

*Note:* Model fit: *R* ^2^ = 0.52; adjusted *R* ^2^ = 0.49; *F*‐statistic = 12.8; overall *p* < 0.001. Regression performed on *n* = 46 participants with available C‐peptide data (complete‐case analysis). The dependent variable is the coefficient of variation (%). *β* coefficients represent the change in CV (percentage points) per unit increase in each predictor, adjusted for all other variables in the model. SE: standard error. *p*‐values in bold indicate significance at *p* < 0.05. Regression assumptions were verified using residual‐versus‐fitted, scale‐location, and normal Q–Q plots; no major violations were identified.

Age approached but did not reach statistical significance (beta 0.21, 95% CI –0.01 to +0.43, *p* = 0.07). Total daily insulin dose was not independently associated with CV (beta 0.04, 95% CI –0.02 to +0.10, *p* = 0.22).

The model explained 52% of the variance in CV (*R* ^2^ = 0.52, adjusted *R* ^2^ = 0.49, *F*‐statistic = 12.8, *p* < 0.001). Inspection of residual plots and influence diagnostics (Cook's distance) did not reveal major violations of regression assumptions or influential outliers.

### 3.4 Dose–Response Relationship Between C‐Peptide and Glycaemic Outcomes

Stratification by C‐peptide category revealed a clear, stepwise dose–response relationship between declining endogenous insulin secretion and worsening glycaemic instability across all primary and secondary outcomes (Table 3, Figure 2).

**TABLE 3:** CGM‐derived glycaemic variability metrics stratified by fasting C‐peptide category.

C‐peptide category	*n*	CV (%)	TIR (%)	TBR (%)	Mean glucose (mg/dL)
Low: C‐peptide < 0.2 ng/mL (near‐complete beta‐cell depletion)	20	44.8 (8.4)	42.1 (12.3)	13.6 (9.7)	182.4 (35.2)
Middle: C‐peptide 0.2–1.0 ng/mL (partial beta‐cell function)	13	38.1 (7.9)	58.4 (14.1)	10.2 (8.1)	155.3 (32.1)
High: C‐peptide > 1.0 ng/mL (preserved beta‐cell function)	13	32.2 (8.2)	65.8 (16.8)	7.8 (7.3)	138.7 (28.9)
*p* for trend		< 0.001	< 0.001	**0.01**	< 0.001

*Note:* Data are presented as mean (SD). C‐peptide categories were pre‐specified using clinical cut‐offs and applied within the 46 participants with available C‐peptide data. *p* for trend was assessed by the Jonckheere–Terpstra test for ordered alternatives across categories. The low category (C‐peptide < 0.2 ng/mL) represents near‐complete beta‐cell depletion, predominantly comprising participants with established Type 1 diabetes. The dose–response pattern across categories was consistent across all four CGM metrics, supporting a biologically coherent and graduated relationship between residual insulin secretion and glycaemic stability.

Abbreviations: CV: coefficient of variation; TBR: time‐below‐range (< 70 mg/dL); TIR: time‐in‐range (70–180 mg/dL).

**FIGURE 2:**
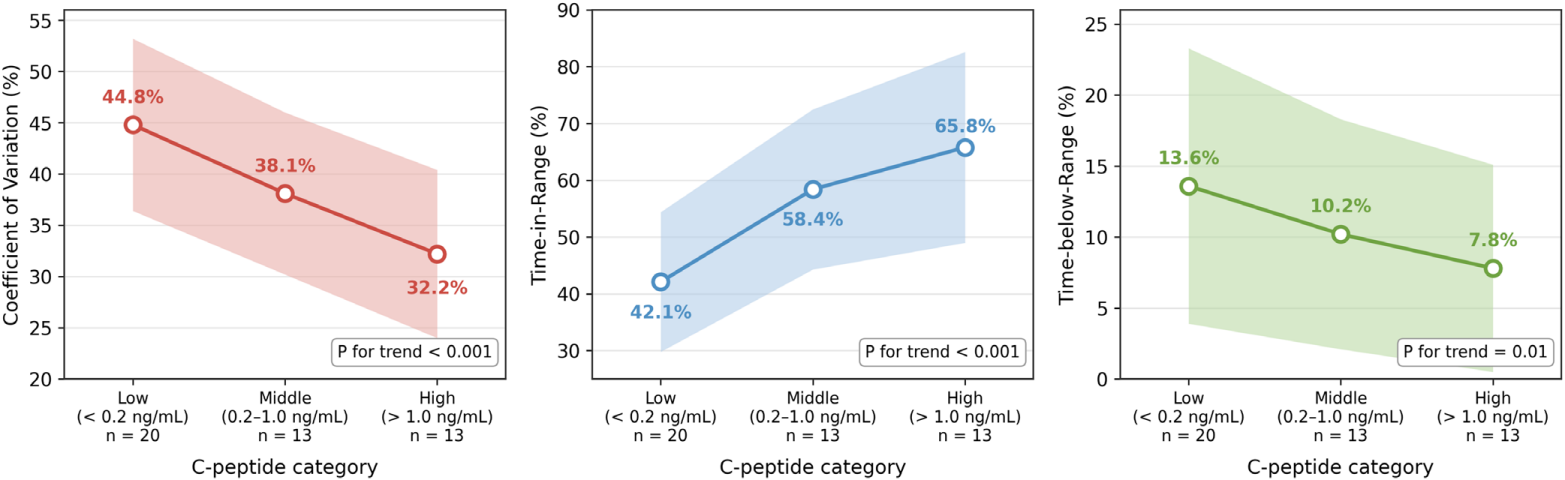
Stepwise dose–response relationship between C‐peptide categories and CGM‐derived glycaemic variability metrics. Line plots showing mean values of coefficient of variation (CV, %), time‐in‐range (TIR, %), and time‐below‐range (TBR, %) across ascending C‐peptide categories (Low: < 0.2 ng/mL, *n* = 20; Middle: 0.2–1.0 ng/mL, *n* = 13; High: > 1.0 ng/mL, *n* = 13). Shaded bands represent ±1 standard deviation. *p* for trend values were assessed by the Jonckheere–Terpstra test. A consistent, graduated improvement in all three metrics with increasing C‐peptide is evident, supporting a dose–response relationship between residual beta‐cell function and glycaemic stability. CGM, continuous glucose monitoring; CV, coefficient of variation; TIR, time‐in‐range; TBR, time‐below‐range.

Participants in the lowest C‐peptide category (below 0.2 ng/mL, representing near‐complete beta‐cell depletion, *n* = 20) had a mean CV of 44.8% (SD 8.4) and TIR of 42.1% (SD 12.3). Those in the highest category (above 1.0 ng/mL, representing relatively preserved beta‐cell function, *n* = 13) achieved a mean CV of 32.2% (SD 8.2) and TIR of 65.8% (SD 16.8). The differences across categories were statistically significant for CV (*p* for trend < 0.001, Jonckheere–Terpstra test), TIR (*p* for trend < 0.001), and mean glucose (*p* for trend < 0.001).

TBR was nearly doubled in the lowest compared with the highest category (13.6% vs. 7.8%, *p* for trend = 0.01), demonstrating that hypoglycaemia burden, not merely hyperglycaemia, tracked inversely with C‐peptide in this cohort.

### 3.5 Sensitivity Analyses

In sensitivity analyses restricted to participants with Type 1 diabetes only (30 of the 46 with available C‐peptide) and to those with near‐complete CGM adherence (at least 10 active CGM days, 43 of the 46 with available C‐peptide), the mean CV and the direction and magnitude of associations between diabetes type, C‐peptide, and CV were consistent with the primary analysis, supporting the robustness of the main findings across clinically relevant subgroups.

## 4 Discussion

In this prospective study of 97 children and adolescents with diabetes in Bangladesh, glycaemic variability substantially exceeded international consensus targets, with nearly 60% of participants failing to achieve the recommended CV of 36% or below. Mean CV was 39.4%, markedly higher than the 32%–37% range reported in well‐resourced paediatric diabetes cohorts from high‐income countries [14, 19, 35, 36]. Type 1 diabetes and lower fasting C‐peptide were independent predictors of elevated CV in multivariable regression, with a clear dose–response relationship across C‐peptide categories. To our knowledge, this represents the first systematic characterisation of glycaemic variability burden using blinded professional CGM in a mixed Type 1 and Type 2 paediatric diabetes cohort from South Asia and the first study to quantify the independent association between fasting C‐peptide and CV in this population.

The observed mean CV of 39.4% is notable not only because it exceeds the international consensus target of 36% [13, 37], but because it was obtained with blinded professional CGM rather than with real‐time personal devices. Real‐time CGM visibility prompts reactive behavioural responses (corrective insulin doses, dietary adjustments, activity modifications) that attenuate observed variability during the monitoring period itself [37, 38, 39, 40]. By using blinded sensors, we removed the behavioural feedback inherent to real‐time devices which typically masks true glycaemic fluctuation, thereby obtaining a less confounded estimate of day‐to‐day variability than most published comparators. This suggests that the glycaemic instability gap between this cohort and high‐income country benchmarks is likely understated.

The elevated variability likely reflects a combination of factors specific to resource‐limited diabetes care: limited access to rapid‐acting and long‐acting analogue insulin formulations, inconsistent meal timing, infrequent clinical contact, and practical barriers to rapid insulin dose adjustment [41]. The finding that 76.5% of participants with Type 1 diabetes exceeded the CV target compared with 24.1% of those with Type 2 diabetes is consistent with the biological distinction between complete and partial beta‐cell failure and aligns with published series from high‐income countries showing that Type 1 diabetes carries substantially greater glycaemic instability [13, 42, 43, 44]. However, the absolute CV values in both subgroups are higher than typically reported (41.9% in Type 1 diabetes and 33.3% in Type 2 diabetes), and even the Type 2 diabetes subgroup, which retained meaningful C‐peptide secretion, showed a mean CV exceeding values reported in comparably managed high‐income country Type 2 cohorts [13, 45, 46, 47]. This suggests that the resource‐limited environment imposes a variability burden that operates across diabetes types and is not fully explained by beta‐cell status alone.

The independent association between fasting C‐peptide and CV (each 1 ng/mL increase in C‐peptide associated with a 1.94 percentage point reduction in CV after adjustment for diabetes type, age, and insulin dose) extends the existing literature in two important directions. First, it confirms in a South Asian paediatric population a relationship demonstrated primarily in adult and high‐income country paediatric cohorts, suggesting that the glycaemic‐stabilising effect of residual endogenous insulin secretion is consistent across ethnicity, socioeconomic context, and diabetes management environment [27, 48, 49]. Second, it demonstrates that this association is detectable using a single fasting C‐peptide measurement obtained in routine clinical biochemistry, a finding with direct relevance for settings where sophisticated beta‐cell function testing is unavailable [21, 50, 51]. The mechanistic basis is biologically coherent: preserved endogenous insulin secretion provides meal‐responsive, physiologically titrated insulin delivery that no exogenous regimen fully replicates, and intact counter‐regulatory glucagon responses buffer against hypoglycaemic excursions, collectively dampening the amplitude of glucose oscillations [23, 52, 53].

The dose–response relationship across C‐peptide categories strengthens this interpretation. Participants in the lowest category (C‐peptide below 0.2 ng/mL, representing near‐complete beta‐cell depletion) had a mean CV of 44.8%, TIR of only 42.1%, and TBR of 13.6%, indicating that both hyperglycaemic and hypoglycaemic burden were simultaneously elevated in the group with the most severe endogenous insulin deficiency [54, 55]. Those in the highest category achieved a mean CV of 32.2% and TIR of 65.8%, approaching international guideline targets despite the absence of advanced insulin delivery technology or structured real‐time CGM use [54, 56]. The fact that TBR was nearly doubled in the lowest compared with the highest category is clinically important: it indicates that variability in this cohort is not merely a hyperglycaemia story, and that hypoglycaemia risk (acutely dangerous in a setting where emergency glucose access may be limited) tracks inversely and meaningfully with C‐peptide. These findings corroborate recent evidence that residual C‐peptide secretion functions as a protective marker for minimising time spent in hypoglycemia by mitigating glucose instability, thereby enabling the pursuit of more ambitious glycaemic goals with an improved safety profile [23, 55, 57].

### 4.1 Clinical Implications

These findings suggest four implications for diabetes care in resource‐limited settings, each of which should be read as provisional given that C‐peptide was available for fewer than half of the cohort. First, our data raise the possibility that fasting C‐peptide, measured at diagnosis or at annual review, could help identify children at higher risk of elevated glycaemic variability. In our cohort, participants with C‐peptide below 0.2 ng/mL (near‐complete beta‐cell depletion) had higher CV (mean 44.8%) and greater time below range (13.6%), and such patients might benefit from prioritised access to blinded professional CGM, more frequent clinical contact, or structured hypoglycaemia avoidance counselling [21, 23, 58]. Whether C‐peptide can usefully guide the allocation of scarce monitoring resources in this way is a hypothesis that would need testing in larger studies before it could be recommended for practice.

Second, the finding that approximately 60% of participants failed to meet CV targets underscores the need to translate CGM data into actionable clinical decisions. Clinics in low‐ and middle‐income countries adopting professional CGM should develop structured protocols for variability‐focused review and insulin dose optimization following each sensor download. Variability reduction (not merely HbA1c reduction) should be an explicit treatment goal, with target CV of 36% or below articulated to patients and families.

Third, the similarity in TBR across both diabetes types (10.9% vs. 10.1%, *p* = 0.73) indicates that hypoglycaemia is not a concern confined to Type 1 diabetes in this cohort, and that variability management and hypoglycaemia avoidance should be explicit treatment goals across the full paediatric diabetes population.

Fourth, if the association we observed is confirmed, low‐cost C‐peptide testing could be worth evaluating as part of routine clinical assessment where feasible. A single fasting C‐peptide measurement costs approximately 5–10 USD in Bangladesh, so the practical barrier to such evaluation is low, but its value for guiding variability‐focused care remains to be established rather than assumed.

### 4.2 Strengths and Limitations

Key strengths include the prospective design with blinded professional CGM (which eliminates the behavioural modification inherent to real‐time devices and provides a more representative estimate of true glycaemic variability [18, 19]), the inclusion of both Type 1 and Type 2 diabetes across a broad paediatric age range, concurrent clinical and biochemical data collection, and the genuinely novel low‐ and middle‐income country South Asian context. The multivariable model demonstrated robust explanatory power (*R* ^2^ = 0.52), and sensitivity analyses restricted to participants with Type 1 diabetes only and to those with near‐complete CGM adherence yielded consistent findings.

Limitations merit candid acknowledgement. The single‐centre design at a national tertiary referral facility means that participants were engaged in specialist diabetes care and may not represent the broader population of children with diabetes in Bangladesh, particularly those with limited access to structured follow‐up. The 14‐day CGM window does not capture seasonal fluctuations, illness‐related glucose excursions, or longer‐term variability patterns. Fasting C‐peptide was available in only 47.4% of participants, necessitating complete‐case analysis for the multivariable model. Although patients with and without C‐peptide data did not differ significantly on primary outcomes, residual selection bias cannot be excluded. Because diabetes type was classified partly on the basis of fasting C‐peptide, and autoantibody testing was not available, some misclassification between Type 1 and Type 2 diabetes is possible, and the overlap between this classifier and the C‐peptide exposure should be kept in mind when interpreting the regression. We did not collect data on dietary intake, socioeconomic status, or specific insulin formulations (rapid‐acting vs. regular, analogue vs. human insulin), all of which are plausible mediators of variability in this setting. Finally, because both fasting C‐peptide and glycaemic variability were assessed at a single time point, the analysis is cross‐sectional and precludes causal or temporal inference; the study is prospective in its data collection but not longitudinal in design. Longitudinal studies tracking how C‐peptide decline during the honeymoon phase of Type 1 diabetes relates to evolving variability burden would substantially strengthen the mechanistic argument.

Prospective multicentre studies from South Asia and sub‐Saharan Africa are needed to define population‐level variability benchmarks appropriate for low‐ and middle‐income country paediatric diabetes cohorts, and to determine whether C‐peptide‐stratified variability risk translates into differential rates of acute complications and long‐term microvascular outcomes. Intervention studies evaluating whether variability‐targeted clinical protocols (including structured CGM data review, C‐peptide‐informed insulin titration, and hypoglycaemia avoidance education) reduce CV and TBR in resource‐limited settings would provide the evidence base for regional guideline development. Cost‐effectiveness analyses comparing professional CGM‐based variability monitoring against standard HbA1c‐only care in low‐ and middle‐income countries would inform resource allocation decisions at the health system level.

## 5 Conclusion

Glycaemic variability substantially exceeds international consensus targets in children and adolescents with diabetes in Bangladesh, with nearly 60% failing to achieve the recommended CV of 36% or below. Type 1 diabetes was the strongest independent predictor of elevated variability, and the dose‐dependent association between fasting C‐peptide and CV is consistent with residual beta‐cell function being an important factor associated with glycaemic stability in this population. Because fasting C‐peptide was available for fewer than half of the participants, these C‐peptide findings should be regarded as hypothesis‐generating; if confirmed in larger samples, fasting C‐peptide could be a low‐cost, widely available marker to help identify children at higher variability risk in settings where CGM access is constrained. Variability reduction should be an explicit treatment goal across both diabetes types in resource‐limited paediatric diabetes care. Larger multicentre studies from South Asia are needed to establish regionally appropriate benchmarks and evaluate C‐peptide‐informed management strategies.

## Author Contributions

**Ajmina Hasan Flabe:** conceptualization, investigation, writing – original draft, methodology, validation, visualization, writing – review and editing, software, formal analysis, project administration, data curation, resources. **Bedowra Zabeen:** conceptualization, supervision, resources, formal analysis, validation, writing – review and editing. **Shatil Amirat:** methodology, data curation, project administration, writing – review and editing, investigation. **Jing Luo:** conceptualization, funding acquisition, writing – review and editing, validation, supervision, resources.

## Funding

This research received no specific grant from any funding agency in the public, commercial, or not‐for‐profit sectors. The Abbott FreeStyle Libre Pro iQ CGM sensors used in this study were made available through the University of Pittsburgh in association with the HumAn‐1 clinical trial. The funding body had no role in the design of the study, data collection, analysis, interpretation of results, or in writing the manuscript.

## Disclosure

Patient and public involvement: Patients and families attending the BADAS Paediatric Diabetes Care and Research Centre were consulted informally during the planning phase regarding the acceptability of 14‐day blinded CGM monitoring and the burden of study participation. Their feedback informed the decision to conduct CGM during routine clinic visits to minimise additional travel requirements. Patients and members of the public were not formally involved in the design of the study, interpretation of results, or preparation of this manuscript. We plan to disseminate study findings to the BADAS patient community through clinic‐based educational sessions and printed materials in Bengali.

## Ethics Statement

This study was approved by the Ethics Review Committee of the Diabetic Association of Bangladesh (Protocol No. BADAS‐ERC/EC/24/16). The study was conducted in accordance with the Declaration of Helsinki and Good Clinical Practice guidelines. Written informed consent was obtained from all participants aged 18 years and above and from the parents or legal guardians of participants under 18 years of age. Age‐appropriate written assent was additionally obtained from participants aged 7 years and above, in accordance with institutional policy.

## Consent

The authors have nothing to report.

## Conflicts of Interest

The authors declare no conflicts of interest.

## Data Availability

The deidentified dataset generated and analysed during this study is not publicly deposited in a repository but is available from the corresponding author (Ajmina Hasan Flabe; ahflabe1996@gmail.com) on reasonable written request. Requests will be considered provided that data sharing is consistent with the terms of the original ethics approval and participant consent.
